# Supplementary material for: The Effect of a Bacillus Probiotic and Essential Oils Compared to an Ionophore on the Rumen Microbiome Composition of Feedlot Cattle
Source: Animals (Basel). 2023 Sep 15;13(18):2927. doi: 10.3390/ani13182927 (PMC10525249; doi:10.3390/ani13182927)
Supplement: Supplementary file 1 [file animals-13-02927-s001.zip › animals-2574031-supplementary.pdf]

Supplementary Tables

Table S1 The compositional relative abundance (in percentage) of the rumen bacterial/archaeal phyla and families of the control (CON), essential oil (EO), monensin (MON) and probiotic (PRO) groups in the starter, grower, and finisher phases.

Bacterial/Archaeal Taxa	CON (%)	EO (%)	MON (%)	PRO (%)	p-value
Starter					
Euryarchaeota	1.53	1.20	2.50	1.09	0.188
Methanobacteriaceae	1.52	1.13	2.48	1.05	0.146
Methanomassiliicoccaceae	0.01	0.07	0.02	0.04	0.346
Actinobacteria	1.13	0.54	1.61	0.48	0.009*
Bifidobacteriaceae	0.24	0.04	0.18	0.05	0.014*
Coriobacteriaceae	0.90	0.50	1.43	0.43	0.016*
Bacteroidetes	60.98	62.19	63.06	61.80	0.667
Bacteroidaceae	0.13	0.46	0.19	0.39	0.102
Marinilabiliaceae	0.36	0.27	0.80	0.30	0.682
Porphyromonadaceae	7.29	4.15	7.44	5.81	0.395
Prevotellaceae	36.01	44.53	47.52	45.46	0.231
Rikenellaceae	0.16	2.97	0.20	1.47	0.409
Flavobacteriaceae	1.52	1.50	0.72	1.16	0.140
Sphingobacteriaceae	0.22	0.45	0.11	0.29	0.518
Elusimicrobia	0.11	0.07	0.06	0.14	0.283
Fibrobacteres	2.88	3.13	0.77	9.11	0.010*
Firmicutes	29.04	28.77	24.46	22.57	0.306
Clostridiaceae_1	0.23	0.10	0.15	0.50	0.646
Clostridiales_Incertae_Sedis_XI	0.04	0.06	0.01	0.01	0.133
Clostridiales_Incertae_Sedis_XIII	0.20	0.15	0.13	0.12	0.159
Lachnospiraceae	7.90	9.31	6.33	6.04	0.771
Ruminococcaceae	14.58	11.57	13.04	9.91	0.277
Erysipelotrichaceae	2.39	3.70	2.82	3.84	0.897
Veillonellaceae	0.13	0.11	0.57	0.08	0.028**
Proteobacteria	3.40	2.97	7.22	2.88	0.902
Rhodospirillaceae	0.27	0.18	1.37	0.37	0.148
Bdellovibrionaceae	0.26	0.09	0.31	0.16	0.047*
Succinivibrionaceae	0.97	0.38	4.56	0.45	0.039*
Spirochaetes	0.65	0.91	0.15	1.82	0.131
Synergistetes	0.05	0.02	0.04	0.02	0.142
Tenericutes	0.19	0.05	0.08	0.05	0.074**
Grower					

Euryarchaeota	0.21	0.84	0.89	0.78	0.056**
Methanobacteriaceae	0.21	0.84	0.85	0.77	0.058**
Methanomassiliicoccaceae	0.01	<0.01	0.04	0.01	0.020*
Actinobacteria	1.45	1.40	0.40	0.92	0.043*
Bifidobacteriaceae	0.24	0.04	0.18	0.05	0.014*
Coriobacteriaceae	0.90	0.50	1.43	0.43	0.016*
Bacteroidetes	18.63	28.57	33.41	21.12	0.030*
Bacteroidaceae	0.37	0.13	0.42	0.13	0.188
Marinilabiliaceae	0.17	0.13	0.15	0.08	0.862
Porphyromonadaceae	1.73	3.90	3.93	1.66	0.044*
Prevotellaceae	11.03	19.02	24.76	14.77	0.012*
Prolixibacteraceae	0.08	0.10	0.03	0.12	0.186
Rikenellaceae	0.26	0.56	0.17	1.28	0.149
Flavobacteriaceae	0.09	0.14	0.22	0.14	0.265
Sphingobacteriaceae	0.48	0.14	0.13	0.01	0.216
Elusimicrobia	0.10	0.07	0.24	0.17	0.098**
Fibrobacteres	0.25	0.50	1.24	0.11	0.043*
Firmicutes	31.81	38.83	21.90	33.87	0.054**
Clostridiaceae_1	0.10	0.43	0.17	0.07	0.259
Clostridiales_Incertae_Sedis_XIII	0.08	0.07	0.05	0.03	0.169
Eubacteriaceae	0.05	0.06	0.01	0.05	0.035*
Lachnospiraceae	14.77	15.13	6.92	13.13	0.034*
Ruminococcaceae	14.33	20.33	12.43	17.90	0.178
Erysipelotrichaceae	0.35	0.47	0.40	0.29	0.903
Veillonellaceae	0.11	0.17	0.06	0.09	0.075**
Proteobacteria	47.40	29.15	41.51	42.93	0.378
Rhodospirillaceae	0.53	0.47	0.48	0.58	0.845
Bdellovibrionaceae	0.18	0.29	0.74	0.22	0.081**
Succinivibrionaceae	0.14	0.27	0.87	0.33	0.028*
Orbaceae	1.53	0.53	1.37	0.87	0.277
Spirochaetes	0.06	0.57	0.35	0.04	0.097**
Synergistetes	0.05	0.04	0.03	0.05	0.568
Finisher					
Euryarchaeota	0.23	0.46	1.61	1.51	0.102
Methanobacteriaceae	0.22	0.45	1.60	1.48	0.099**
Actinobacteria	0.30	0.34	0.26	0.74	0.084**
Coriobacteriaceae	0.27	0.33	0.25	0.72	0.090**
Bacteroidetes	23.24	28.38	29.75	31.91	0.195
Bacteroidaceae	0.08	0.05	0.06	0.07	0.693
Bacteroidales_incertae_sedis	0.04	0.06	0.07	0.05	0.395

Marinilabiliaceae	0.10	0.09	0.15	0.46	0.029*
Porphyromonadaceae	1.06	1.66	1.91	1.74	0.272
Prevotellaceae	19.70	22.73	24.89	20.61	0.544
Prolixibacteraceae	0.05	0.05	0.02	0.05	0.218
Rikenellaceae	0.18	0.47	0.11	0.18	0.118
Flavobacteriaceae	0.02	0.05	0.01	0.06	0.146
Sphingobacteriaceae	0.23	0.41	0.14	2.05	0.045*
Elusimicrobia	0.07	0.04	0.04	0.14	0.006*
Fibrobacteres	0.20	0.30	0.22	0.44	0.124
Firmicutes	9.86	11.99	17.60	21.03	0.023*
Clostridiaceae_1	0.17	0.06	0.45	0.21	0.241
Clostridiales_Incertae_Sedis_XIII	0.04	0.04	0.03	0.09	0.086**
Eubacteriaceae	0.02	0.03	0.01	0.04	0.121
Lachnospiraceae	4.39	5.29	3.84	7.95	0.035*
Ruminococcaceae	4.38	5.52	12.42	10.62	0.024*
Erysipelotrichaceae	0.19	0.16	0.26	0.51	0.170
Acidaminococcaceae	0.02	0.04	0.02	0.08	0.040*
Veillonellaceae	0.05	0.09	0,10	0.11	0.122
Proteobacteria	66.00	58.23	50.37	44.12	0.058**
Rhodospirillaceae	0.08	0.14	0.05	0.37	0.080**
Bdellovibrionaceae	0.04	0.19	0.08	0.18	0.046*
Succinivibrionaceae	0.35	0.20	4.63	0.22	0.067**
Orbaceae	1.32	1.39	2.25	2.13	0.608
Spirochaetes	0.06	0.21	0.10	0.03	0.502
Synergistetes	0.04	0.04	0.04	0.07	0.032*

* Significance at $p < 0.05$

** Tendency towards significance at $p < 0.10$

Table S2 The relative abundance (in percentage) of the rumen fungal phyla and families in the control (CON), essential oils (EO), monensin (MON) and probiotic (PRO) groups in the starter, grower, and finisher phases.

Fungal Phyla	CON (%)	EO (%)	MON (%)	PRO (%)	p-value
Starter					
Ascomycota	75.99	73.79	73.07	58.50	0.787
Cladosporiaceae	3.93	3.67	4.22	2.41	0.395
Aureobasidiaceae	1.34	1.28	1.53	1.96	0.792
Dothideales_fam_Incertae_sedis	16.51	11.10	12.65	8.33	0.231
Didymellaceae	34.72	36.55	37.79	20.74	0.235
Didymosphaeriaceae	0.69	0.64	0.63	0.74	0.891
Massarinaceae	0.30	0.18	0.15	0.10	0.111

Montagnulaceae	0.56	0.15	0.30	0.11	0.026*
Phaeosphaeriaceae	0.85	1.04	0.53	0.84	0.402
Pleosporaceae	2.13	2.27	2.00	2.02	0.857
Sporormiaceae	0.21	0.16	0.40	0.25	0.157
Aspergillaceae	9.46	11.07	7.42	12.04	0.544
Trichocomaceae	0.23	0.27	0.30	0.27	0.638
Myxotrichaceae	0.24	0.33	0.07	0,23	0.059**
Phaffomycetaceae	0.11	0.13	0.23	0.13	0.866
Plectosphaerellaceae	0.11	0.39	0.22	3.14	0.012*
Hypocreales_fam_Incertae_sedis	0.69	1.29	0.61	0.94	0.303
Nectriaceae	0.74	0.56	1.03	1.20	0.181
Stachybotryaceae	0.26	0.18	0.33	0.23	0.326
Microascaceae	0.35	0.18	0.27	0.19	0.549
Chaetomiaceae	0.64	0.57	0.59	0.70	0.866
Togniniaceae	0.20	0.16	0.11	0.09	0.977
Trichosphaeriaceae	0.26	0.24	0.18	0.28	0.565
Basidiomycota	4.13	3.45	7.30	6.13	0.287
Phallaceae	0.04	0.02	0.21	0.08	0.041*
Cystobasidiomycetes	0.11	0.03	0.11	0.10	0.080**
Sporidiobolaceae	0.08	0.05	0.23	0.14	0.424
Filobasidiaceae	3.60	3.04	6.30	5.34	0.309
Tremellaceae	0.09	0.06	0.16	0.19	0.273
Ustilaginaceae	0,03	0.06	0.11	0.09	0.152
Mortierellomycota	0	0	0.06	0.13	0.542
Mucoromycota	0.39	0.53	0.56	0.68	0.562
Lichtheimiaceae	0.14	0.29	0.19	0.20	0.687
Mucoraceae	0.24	0.23	0.26	0.41	0.177
Neocallimastigomycota	18.80	21.46	18.43	33.75	0.084**
Anthophyta	0.67	0.77	0.57	0.78	0.902
Grower					
Ascomycota	89.50	91.31	89.47	81.31	0.033*
Phaeococcomycetaceae	0.12	0.11	0.07	0.19	0.717
Cladosporiaceae	2.27	1.37	1.32	1.39	0.750
Teratosphaeriaceae	0.28	0.26	0.08	0.29	0.159
Aureobasidiaceae	0.17	0.19	0.30	0.38	0.740
Dothideales_fam_Incertae_sedis	0.54	0.79	1.18	0.98	0.024*
Didymellaceae	8.69	11.96	10.45	12.60	0.782
Didymosphaeriaceae	3.08	3.48	1.86	3.24	0.258
Phaeosphaeriaceae	3.89	5.44	2.40	5.51	0.647
Pleosporaceae	0.85	0.82	0.92	1.24	0.899

Sporormiaceae	0.29	0.42	0.24	0.30	0.326
Teichosporaceae	0.40	0.43	0.15	0.25	0.025*
Tubeufiaceae	0.06	0.26	0.01	0.08	0.063**
Herpotrichiellaceae	0.12	0.16	0.04	0.14	0.204
Aspergillaceae	46.45	37.18	50.65	30.02	0.034*
Trichocomaceae	3.12	4.72	2.12	2.44	0.012*
Myxotrichaceae	2.30	1.07	2.35	1.96	0.202
Debaryomycetaceae	0.20	0.24	0.26	0.32	0.645
Phaffomycetaceae	0.77	0.38	2.94	0.44	0.133
Saccharomycetaceae	0.04	0.06	0.12	0.20	0.287
Chaetosphaeriaceae	0.10	0.15	0.07	0.11	0.732
Diaporthaceae	0.23	0.27	0.16	0.33	0.775
Plectosphaerellaceae	0.30	0.25	0.15	0.08	0.026*
Bionectriaceae	0.21	0.37	0.13	0.22	0.107
Cordycipitaceae	0.13	0.10	0.06	0.13	0.085**
Hypocreaceae	1.24	1.38	1.03	1.21	0.442
Hypocreales_fam_Incertae_sedis	1.71	1.78	1.15	1.43	0.097**
Nectriaceae	2.22	3.10	2.11	2.75	0.792
Stachybotryaceae	0.17	0.16	0.09	0.13	0.048*
Microascaceae	0.16	0.19	0.29	0.17	0.016*
Myrmecridiaceae	0.16	0.25	0.07	0.11	0.030*
Chaetomiaceae	5.97	9.59	4.32	6.98	0.075**
Trichosphaeriaceae	0.61	0.99	0.77	0.98	0.687
Apiosporaceae	0.11	0.13	0.06	0.13	0.248
Basidiomycota	0.97	1.52	1.61	1.49	0.012*
Cystobasidiomycetes	0.18	0.22	0.08	0.26	0.224
Erythrobasidiaceae	0.12	0.14	0.07	0.10	0.088**
Sporidiobolaceae	0.20	0.42	0.39	0.37	0.567
Filobasidiaceae	0.24	0.55	0.90	0.54	0.017*
Mucoromycota	2.10	2.44	2.43	2.78	0.309
Lichtheimiaceae	1.49	1.48	0.59	0.92	0.035*
Mucoraceae	0.52	0.53	1.71	1.68	0.056**
Neocallimastigomycota	6.76	4.24	6.14	13.45	0.544
Anthophyta	0.57	0.37	0.17	0.85	0.019*
Finisher					
Ascomycota	54.27	35.79	43.97	50.60	0.056**
Cladosporiaceae	0.15	0,21	0.14	0.20	0.860
Aureobasidiaceae	1.38	1.34	0.51	0.52	0.012*
Dothideales_fam_Incertae_sedis	0.76	0.92	0.38	0.61	0.274
Didymellaceae	16.59	17.68	9.58	18.63	0.210

Didymosphaeriaceae	0.78	0.51	0.61	1.03	0.051**
Massarinaceae	0.57	0.38	0.56	0.73	0.042*
Phaeosphaeriaceae	0.30	0.29	0.20	0.30	0.146
Aspergillaceae	23.59	4.29	21.59	21.14	0.018*
Trichocomaceae	0.26	0.19	1.24	0.69	0.018*
Debaryomycetaceae	0.19	0.17	0.16	0.16	0.548
Phaffomycetaceae	0.19	0.39	0,24	0,28	0.831
Diaporthaceae	0.23	0.28	0.19	0.16	0.142
Clavicipitaceae	0.15	0.36	0.10	0.18	0.041*
Hypocreales_fam_Incertae_sedis	0.87	0.45	0.53	0.62	0.047*
Nectriaceae	6.98	6.85	4.26	3.73	0.025*
Chaetomiaceae	0.17	0.09	0.27	0.25	0.052**
Basidiomycota	1.16	1.89	0.63	1.52	0.092**
Sporidiobolaceae	0.47	0.79	0.20	0.51	0.082**
Filobasidiaceae	0.37	0.76	0.18	0.63	0.080**
Ustilaginaceae	0.24	0.03	0.14	0.23	0.015*
Mucoromycota	1.48	11.47	0.90	2.09	0.102
Lichtheimiaceae	0.39	0.13	0.10	0.36	0.009*
Mucoraceae	1.08	11.34	0.79	1.73	0.156
Neocallimastigomycota	42.67	47.82	53.94	45.06	0.350
Anthophyta	0.43	3.03	0.56	0.72	0.390

* Significance at $p < 0.05$

** Tendency towards significance at $p < 0.10$
